# Investigating the Relationships Among Gut Microbiota, Inflammatory Cytokines, Cerebrovascular Diseases, and the Mediation Pathways

**DOI:** 10.1155/mi/8623083

**Published:** 2026-02-23

**Authors:** Qi Li, Xinpeng Liu, Xiang Deng, Houxiang Huang, Xinrui Wu

**Affiliations:** ^1^ School of Medicine, Jishou University, Jishou, China, jsu.edu.cn; ^2^ Xiangxi Center for Disease Control and Prevention, Jishou, China

**Keywords:** causal relationships, cerebrovascular disease, gut microbiota, inflammatory cytokines, mediation analysis

## Abstract

**Objectives:**

Cerebrovascular disease (CeVD), the second leading global cause of death, has an incompletely understood pathogenesis. This study aimed to investigate causal relationships among gut microbiota, inflammatory cytokines, and CeVD, specifically examining inflammatory cytokine‐mediated pathways.

**Methods:**

Genome‐wide association study (GWAS) summary statistics for 209 gut microbial taxa, 91 inflammatory cytokines, and three CeVD subtypes were obtained from publicly available datasets. Causal effects were estimated by applying four complementary two‐sample Mendelian randomization (MR) methods. Reverse MR and comprehensive sensitivity analyses were performed to validate the robustness of the findings. Multivariable MR (MVMR) analyses were conducted to account for potential confounders. Mediation analysis was conducted to elucidate the pathways from gut microbiota to CeVD, mediated by inflammatory cytokines.

**Results:**

Our study identified 33 gut microbiota significantly associated with CeVD, including nine with ischemic stroke (IS), 14 with intracerebral hemorrhage (ICH), and 10 with subarachnoid hemorrhage (SAH). Eighteen inflammatory cytokines showed significant associations with different CeVD subtypes. Mediation analysis revealed 10 causal pathways from gut microbiota to CeVD mediated by inflammatory cytokines; among these, three inflammatory cytokines mediated more than two pathways.

**Conclusions:**

This study demonstrated that inflammatory cytokines mediated the gut microbiota–CeVD causal pathway through genetic evidence, elucidating novel disease mechanisms, thereby providing actionable insights for developing CeVD prevention and treatment strategies.

## 1. Introduction

Cerebrovascular diseases (CeVDs) encompass a variety of conditions resulting from abnormalities in the blood vessels, which disrupt blood flow to the brain and cause brain tissue damage. The primary CeVD subtypes, accounting for 87% of global stroke cases, are ischemic stroke (IS), intracerebral hemorrhage (ICH), and subarachnoid hemorrhage (SAH) [1]. According to the Global Burden of Disease Study, CeVD ranks as the second leading global cause of mortality, responsible for 6.6 million annual deaths [2]. With the aging global population, the incidence of CeVD is rising, posing an increasing threat to public health and exerting significant pressure on healthcare systems [3]. Despite the growing burden, the precise mechanisms underlying CeVD, along with effective prevention and treatment strategies, remain poorly understood.

The gut microbiota constitutes a metabolically active, taxonomically diverse ecosystem colonizing the human gastrointestinal tract. Research has shown a bidirectional relationship between the brain and the gut microbiota, facilitated by the microbiota–gut–brain axis [4, 5]. Gut microbiota and their metabolites contribute to the development of IS by modulating critical processes, including oxidative stress, apoptosis, neuroinflammation, and other related pathways [6]. Changes in microbial populations, such as the increase in *Enterococcus* and the decrease in *Prevotella*, potentially impact recovery outcomes in ICH patients [7]. Furthermore, animal studies have shown that gut microbiota can affect early brain injury after SAH by regulating neutrophil infiltration [8].

Inflammatory cytokines are signaling molecules produced by immune cells that play a crucial role in regulating inflammation and immune responses [9]. Numerous studies have demonstrated the strong association between inflammatory cytokines and CeVD. These factors can activate immune cells, leading to the release of various mediators that cause endothelial damage, disrupt the blood–brain barrier, and lead to cerebral edema, ultimately worsening the condition [10]. Chen et al. [11] found that chemokine‐like factor triggers downstream signaling pathways by binding to its receptor, thus influencing the development and progression of CeVD. A cross‐sectional study involving 3052 participants indicated that the elevated level of systemic immune‐inflammation index correlates with a higher risk of CeVD [12].

The complex interplay within the human body makes it difficult to measure confounding factors in studies involving gut microbiota, inflammation, and CeVD. Furthermore, establishing the temporal order of exposure and outcome remains challenging. To address these issues, the application of Mendelian randomization (MR) methods is essential. MR is an epidemiological method leveraging germline genetic variants as instrumental variables (IVs) to estimate causal exposure–outcome effects under the core IV assumptions (relevance, independence, and exclusion restriction), while accounting for potential pleiotropy [13]. Unlike traditional observational studies, MR leverages the fact that genetic variants are fixed at birth, thereby minimizing biases from unmeasured confounding factors and reverse causality [14]. In addition to MR, mediation analysis is employed to assess how the effect of the exposure on the outcome is transmitted through the mediator. This method helps to elucidate the underlying pathways and relationships between variables [15]. In this study, we utilized publicly available genome‐wide association study (GWAS) summary data to conduct both MR and mediation analyses. Our goal was to evaluate the causal relationship between the gut microbiota, inflammatory cytokines, and CeVD and to identify potential pathways through which gut microbiota influences CeVD via inflammatory cytokines.

## 2. Materials and Methods

### 2.1. Study Design

**Figure 1:**
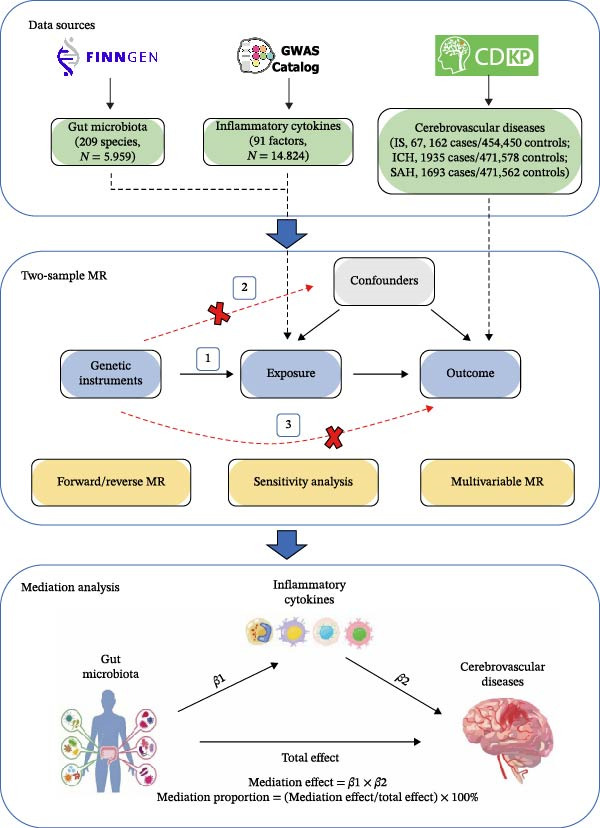
Flowchart of this study. The MR approach is based on three fundamental assumptions: (1) genetic instruments must have a strong association with exposure; (2) genetic instruments must be independent of confounders that influence both exposure and outcome; and (3) genetic instruments should influence the outcome solely through exposure.

Figure 1 presents the flowchart of this study. We extracted publicly available GWAS summary statistics for gut microbiota, inflammatory cytokines, and CeVD subtypes. Two‐sample MR analyses, including forward/reverse and multivariable MR (MVMR) analyses, were conducted to assess causal relationships among these traits. Ultimately, mediation analysis was employed to uncover the mediation role of inflammatory cytokines in the gut microbiota–CeVD association. Our MR study adhered to the STROBE‐MR guidelines (Supporting Information 1: Table S1) [16].

### 2.2. Data Sources

GWAS summary statistics for gut microbiota composition were derived from the FINRISK 2022 cohort (*n* = 5959, European‐ancestry), with genomic and metagenomic data generated through shotgun fecal sequencing [17]. High‐quality genotypes (imputation quality score >0.8) and taxonomic profiles were generated, with almost 8 million genetic variants analyzed after stringent quality control (QC). Taxonomic annotation utilized the GTDB database as a reference [18], profiling 209 species‐level taxa across six taxonomic ranks (phylum to species) using MetaPhlAn4 with default parameters.

The GWAS datasets for each inflammatory cytokine are publicly accessible in the GWAS Catalog, with registration numbers ranging from GCST90274758 to GCST90274848 [19], generated using the Olink Target 96 Inflammation v3 panel. These data derive from the INTERVAL cohort (*n* = 14,824, European‐ancestry), comprising 11 independent studies with stringent QC.

The GWAS datasets for CeVDs, serving as outcomes, were obtained from the Cerebrovascular Diseases Knowledge Portal (CDKP) platform [20]. Specifically, the multiancestry GWAS involving 521,612 individuals (67,162 cases and 454,450 controls) provided genetic variations for IS. Genetic variants for ICH and SAH were collected from a meta‐analysis of UK Biobank and FinnGen cohorts, including 473,513 European‐ancestry subjects (1935 cases and 471,578 controls) and 473,255 subjects (1693 cases and 471,562 controls), respectively.

### 2.3. Selection of IVs

We selected single nucleotide polymorphisms (SNPs) that are strongly associated with gut microbiota and inflammatory cytokines as IVs, using a significance threshold of *p*  < 1e–05 [21]. To minimize potential bias from linkage disequilibrium, we applied the clumping procedure in PLINK software, with a cutoff of *r* ^2^ <0.001 and a window size of 10,000 kb. We selected proxy SNPs (*r* ^2^ > 0.8) from the samples of the 1000 Genomes Project, excluding any SNP with genome‐wide significance for the outcome (*p*  < 5e–08). Furthermore, to avoid weak instrument bias, we retained IVs with *F*‐statistics greater than 10 [22].

### 2.4. MR Analyses

In this study, we conducted MR analysis to investigate the potential causal relationship between gut microbiota, inflammatory cytokines, and CeVD. The primary analysis was conducted using inverse‐variance‐weighted (IVW) meta‐regression, which aggregates Wald ratios from individual IVs under a fixed intercept of zero [23]. Assuming the absence of horizontal pleiotropy, this method yields the most precise and impartial causal estimates [24]. To reduce the likelihood of Type I errors from multiple comparisons, we implemented the false discovery rate (FDR) correction on the primary IVW results. Additionally, we applied several other MR methods, including maximum likelihood (MaxLik), MR‐robust adjusted profile score (MR‐RAPS), and Bayesian weighted MR (BWMR). The MaxLik method typically yields smaller standard errors compared to the IVW method, owing to the properties of MaxLik estimation [25]. The MR‐RAPS approach provides stable results in the presence of heterogeneity, multiple comparisons, and weak IVs [26]. BWMR is well‐suited for two‐sample MR, as it can estimate weak effects due to polygenicity and detect outlier IVs [27]. Consistency across different methods supports the robustness of the observed causal effect.

To improve the accuracy of our estimates, we performed the Cochran *Q* test to assess heterogeneity. In addition, we used the MR‐Egger intercept and MR‐PRESSO global test to evaluate horizontal pleiotropy [28]. MVMR analyses were conducted, accounting for potential confounding factors that may affect the outcome, including blood pressure, smoking, and alcohol drinking.

### 2.5. Mediation Analyses

To investigate the mechanisms through which gut microbiota influences CeVD, we performed mediation analyses, using inflammatory cytokines as the mediator [29]. The first step involved estimating the causal effect of genetically determined gut microbiota on inflammatory cytokines (*β*1). Subsequently, we estimated the causal effect of inflammatory cytokines on CeVD subtypes (*β*2). We calculated the mediation effect, direct effect, and mediation proportion using the following equations:

mediation effect=β12100×β, direct effect=total effect−mediation effect, and mediation proportion=mediation effect/total effect×%.

The delta method was applied to derive standard errors from the two‐sample MR analyses.

## 3. Results

### 3.1. Causal Relationships Between Gut Microbiota and CeVD

**Figure 2:**
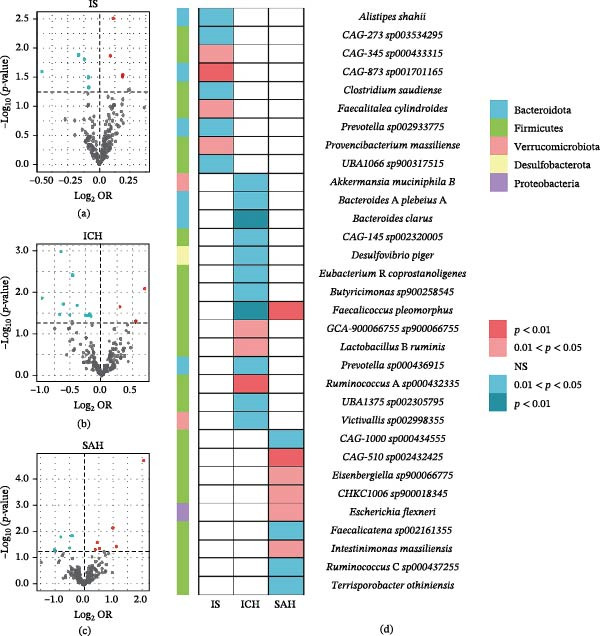
Causal estimates of gut microbiota on CeVD. (a–c) Volcano plots of the IVW MR for the associations between gut microbiota and CeVD subtypes. (d) Heatmap of the 32 gut microbiota species that showed a causal association with IS, ICH, and SAH at nominal significance (*p*_IVW_ < 0.05).

IVW analysis revealed 33 significant causal associations between gut microbiota species and CeVD at *p*  < 0.05. These relationships corresponded to five different gut microbiota phyla. Notably, we observed significant positive associations between the increase in the abundance of *Faecalitalea cylindroides* (OR, 1.141; 95%CI, 1.013–1.285; *p* = 0.030), *Provencibacterium massiliense* (OR, 1.137; 95%CI, 1.012–1.279; *p* = 0.031), *CAG-345 sp000433315* (OR, 1.064; 95%CI, 1.013–1.117; *p* = 0.014), *CAG-873 sp001701165* (OR, 1.082; 95%CI, 1.027–1.140; *p* = 0.003), and the elevated risk of IS. Conversely, genetically increased levels of *Alistipes shahii* (OR, 0.942; 95%CI, 0.892–0.995; *p* = 0.032), *CAG-273 sp003534295* (OR, 0.943; 95%CI, 0.889–0.999; *p* = 0.048), *Clostridium saudiense* (OR, 0.920; 95%CI, 0.860–0.985; *p* = 0.016), *Prevotella sp002933775* (OR, 0.891; 95%CI, 0.812–0.976; *p* = 0.015), and *UBA1066 sp900317515* (OR, 0.726; 95%CI, 0.548–0.962; *p* = 0.026) were associated with the protective effect on IS. We also explored the causal relationships between 14 gut microbiota species and ICH, as well as 10 species and SAH, respectively. However, these associations lost their significance after adjusting for multiple comparisons (*q* > 0.1). The median *h* ^2^ estimates for gut microbial taxa were 0.31%, while the *F*‐statistics for these results ranged from 20.903 to 25.039, suggesting the absence of bias due to weak IVs. Moreover, consistent causal associations between gut microbiota and CeVD risk were identified using different MR methods (Figure 2, Supporting Information 1: Table S2).

Cochran’s *Q* test demonstrated no evidence of heterogeneity among IVs, while the MR‐Egger intercept and MR‐PRESSO global test collectively confirmed the absence of directional horizontal pleiotropy (*p* > 0.05, Supporting Information 1: Table S3). The leave‐one‐out analysis further demonstrated that no single IV had a substantial effect on the results when excluded (Supporting Information 2: Figures S1–S3).

**Figure 3:**
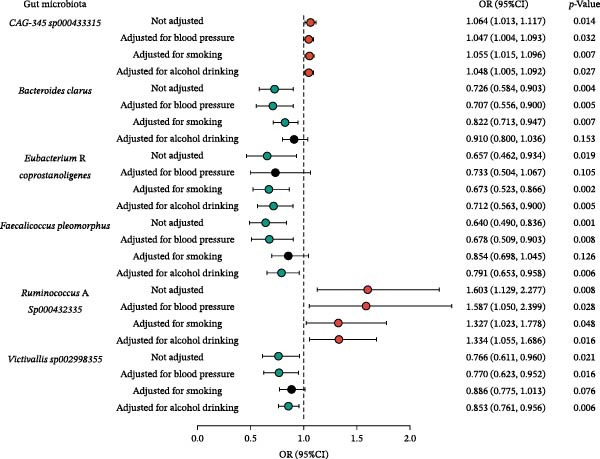
Forest plot of causal estimates of gut microbiota on CeVD from multivariable MR analysis. Six gut microbiota species remain statistically significant after adjusting for two or more confounding factors.

Reverse MR revealed no causal effects of CeVD on gut microbiota composition (FDR‐corrected *p*  > 0.05), refuting bidirectional causality (Supporting Information 1: Table S4). Sensitivity analyses confirmed robustness across all four MR methods (Supporting Information 1: Table S5). Additionally, MVMR analysis identified six gut microbiota species that were significantly associated with CeVD risk after controlling for more than two confounding factors. Detailed results from the MVMR analysis for other gut microbiota and CeVD subtypes are provided in Figure 3 and Supporting Information 1: Table S6.

### 3.2. Causal Relationships Between Inflammatory Cytokines and CeVD

**Figure 4:**
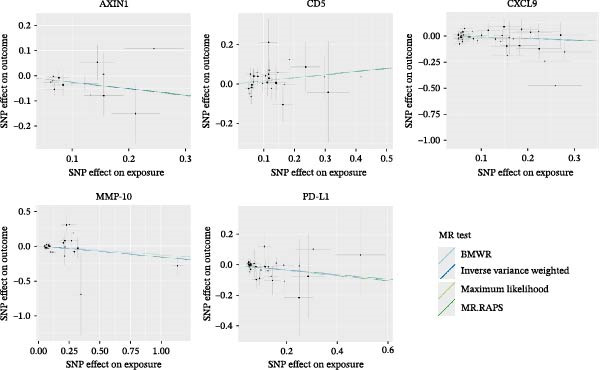
Scatter plots for causal associations between inflammatory cytokines and ICH.

Based on the results obtained using the IVW method, we identified seven inflammatory cytokines associated with IS, five with ICH, and six with SAH. For instance, increased abundance of CD5 (OR, 1.169; 95%CI, 1.001–1.365; *p* = 0.049) was associated with a higher risk of ICH. In contrast, higher levels of AXIN1 (OR, 0.777; 95%CI, 0.618–0.978; *p* = 0.031), CXCL9 (OR, 0.862; 95%CI, 0.744–0.998; *p* = 0.047), matrix metalloproteinase‐10 (MMP‐10; OR, 0.853; 95%CI, 0.739–0.985; *p* = 0.031), and programmed cell death‐ligand 1 (PD‐L1; OR, 0.860; 95%CI, 0.744–0.994; *p* = 0.041) were found to be protective against ICH. The median *h* ^2^ estimates were 5.7%, and the *F*‐statistics for these results ranged from 20.629 to 59.237, suggesting the absence of bias due to weak IVs (Figure 4, Supporting Information 1: Table S7).

The leave‐one‐out analysis confirmed that no IVs significantly affected the results (Supporting Information 2: Figures S4–S6). However, following sensitivity analyses, we excluded five inflammatory cytokines—four related to IS and one to SAH—due to observed heterogeneity in the IVs and evidence of directional horizontal pleiotropy (Supporting Information 1: Table S8). All methods yielded no evidence of reverse causal relationships (*p* > 0.05, Supporting Information 1: Table S9, S10). Furthermore, sensitivity analyses confirmed the robustness of null reverse MR results (Supporting Information 1: Table S10).

### 3.3. Mediation Analysis

Mediation analysis quantified the proportion of the gut microbiota–CeVD causal pathway mediated by inflammatory cytokines (counterfactual‐based structural equation modeling, 95% *CI*), specifically testing the indirect effect: gut microbiota → cytokines → CeVD. The analysis incorporated only microbial taxa (*n* = 209) and cytokines (*n* = 91) demonstrating causal effects on CeVD in primary MR analyses, excluding nonsignificant mediators.

In summary, we identified 10 mediating relationships. The pathway from the gut microbiota species *Akkermansia muciniphila* B to ICH was mediated by MMP‐10 and PD‐L1, accounting for 7.46% and 7.04% of the effect, respectively. Additionally, three inflammatory cytokines—MMP‐10, PD‐L1, and TNF—mediated several relationships. For example, MMP‐10 levels mediated the association between *A. muciniphila* B, *GCA-900066755 sp900066755*, and *Prevotella sp000436915* with ICH, with mediation effects of 7.46%, 8.08%, and 13.96%, respectively (Figure 5, Supporting Information 1: Table S11).

Figure 5The 10 causal pathways (a–j) from gut microbiota to CeVD, mediated by circulating inflammatory cytokines.

**Figure:**
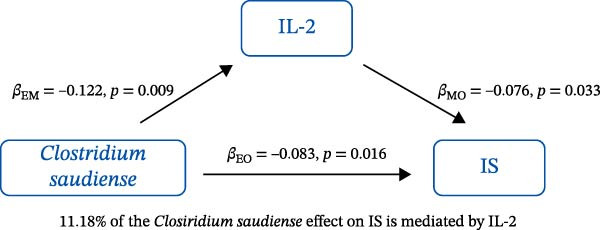
(a)

**Figure:**
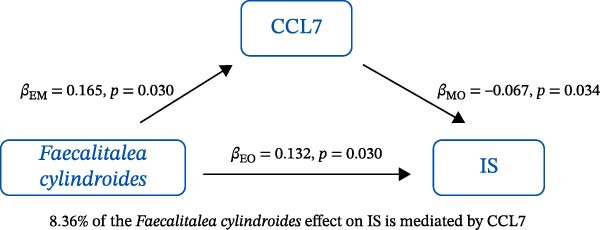
(b)

**Figure:**
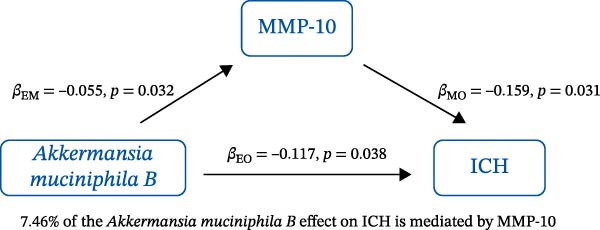
(c)

**Figure:**
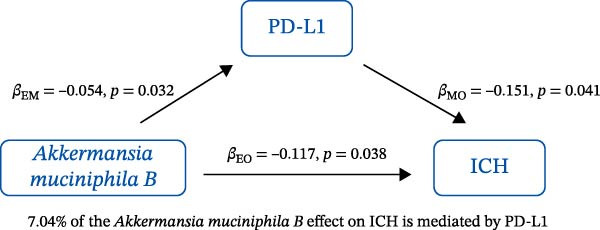
(d)

**Figure:**
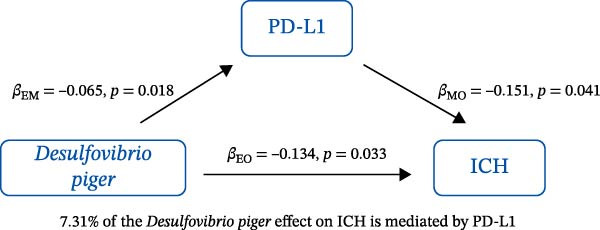
(e)

**Figure:**
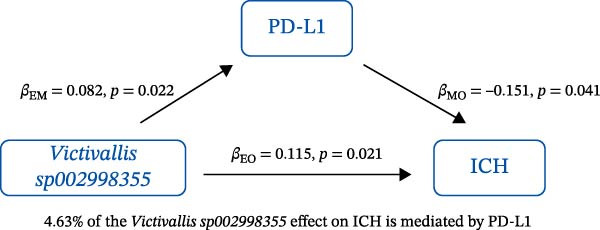
(f)

**Figure:**
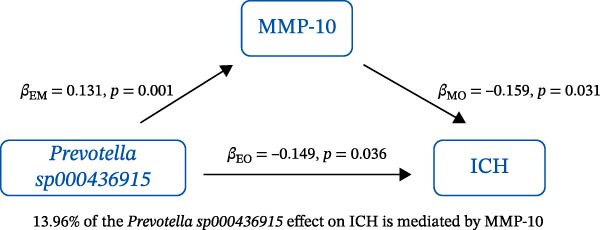
(g)

**Figure:**
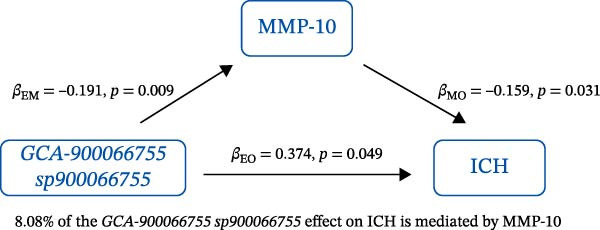
(h)

**Figure:**
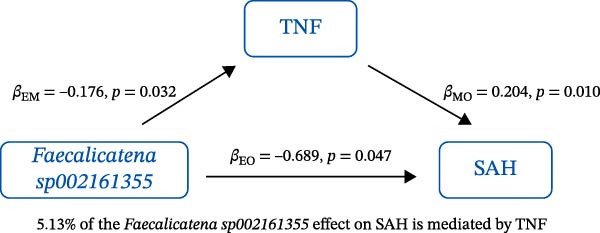
(i)

**Figure:**
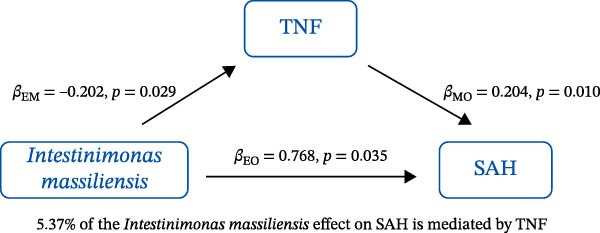
(j)

## 4. Discussion

In this study, we identified causal effects of 33 microbial taxa on CeVD through MR analyses. After adjusting for more than two confounding factors, the causal associations of six gut microbiota species remained statistically significant. Eighteen inflammatory cytokines demonstrated subtype‐specific causal effects. Mediation analysis quantified that inflammatory proteins mediated the gut microbiota–CeVD causal pathways, with *Prevotella sp000436915* → MMP‐10 → ICH constituting the dominant indirect effect.

*Faecalicoccus pleomorphus*, a species within the *Faecalicoccus* genus, was first isolated from the cecal contents of chicken [30]. The Firmicutes phylum can positively influence health through the dietary fiber–Firmicutes–host axis, primarily by enhancing the absorption of dietary fiber and supporting intestinal health and function [31]. Additionally, Firmicutes has been shown to increase interleukin‐10 (IL‐10) and decrease IL‐17 levels [31]. IL‐10, an anti‐inflammatory factor, plays a protective role against IS, whereas IL‐17, a pro‐inflammatory factor, promotes the development of IS [32]. Interestingly, our findings suggested that *F. pleomorphus* may act as a protective factor for ICH at the species level. However, its increased abundance appeared to pose a significant risk for SAH. We hypothesized that this discrepancy may stem from differences in the underlying pathogenic mechanisms, which warrants further investigation to clarify the causal relationship.

The microbiota genus *Prevotella* includes over 50 species, many of which are closely related to human health [33], though its role in disease remains controversial. Numerous studies have shown elevated levels of *Prevotella* in inflammatory diseases such as periodontitis and rheumatoid arthritis [34, 35]. Li et al. [36] detected an increased abundance of *Prevotella* in hypertensive populations, which is a known risk factor for CeVD. A population‐based study also observed *Prevotella* enrichment in patients with IS [37], although findings by Park et al. [38] contradicted this observation. Additionally, Filippis et al. [39] found that individuals with vegetarian diets exhibited higher *Prevotella* abundance, which may contribute to improved glucose metabolism by promoting glycogen storage, thus offering protection to CeVD [40]. Notably, our study found that *Prevotella sp002933775* has a protective effect on IS, while *Prevotella sp000436915* appears to decrease the risk of SAH. These results emphasized the need to investigate different *Prevotella* species in relation to specific disease subtypes to better understand the underlying mechanisms from the perspective of gut microbiota.

Our study explored negative causal relationships between MMP‐10, PD‐L1, and ICH, respectively. MMP‐10 has been shown in the mouse model to significantly reduce intracranial reperfusion time and infarct size, with shorter bleeding times and fewer intracranial hemorrhagic events compared to tissue plasminogen activator [41]. This finding was consistent with research by Roncal et al. [42]. Additionally, Navarro‐Oviedo et al. [43] found that MMP‐10 treatment reduced the expression of Nox2, a marker of oxidative stress, and decreased systemic thrombin activation. PD‐L1, a key immune regulator, has been shown to alleviate secondary brain injury following brain hemorrhage, including neuronal cell death, neurodegeneration, and inflammation [44]. The animal experiment also demonstrated that PD‐L1 improves the inflammatory environment, strengthens the blood–brain barrier, and reduces brain edema as well as hemorrhage volume in ICH model mice [45]. Furthermore, Liu et al. [46] found that PD‐1/PD‐L1 modulation of microglial differentiation, suppression of pro‐inflammatory cytokines, and improvement in inflammatory cell infiltration can mitigate secondary brain injury in rats with acute brain hemorrhage. The evidence presented above supports our results that MMP‐10 and PD‐L1 are protective factors for the occurrence of ICH.

In our study, we identified mediating relationships in which pathways from the gut microbiota species *A. muciniphila* B to ICH were mediated by MMP‐10 and PD‐L1, contributing 7.46% and 7.04% to the effect, respectively. Although the individual contributions of each mediator (MMP‐10: 7.46%; PD‐L1: 7.04%) appear limited, these results align with existing research on the complex role of gut microbiota in disease. Recent mediation analysis studies showed that the microbiota mediates the risk of hypertension through a single inflammatory factor by 4.0%−7.9% [47] and the risk of Alzheimer’s disease through immune/vascular factors by 6.9%−14.3% [48]. This suggests that ~7% of the effect is representative of the “microbiota–host–disease” pathway. More importantly, when multiple mediators are considered together, the total mediated effect can be substantially enhanced [48]. This highlights the potential of MMP‐10 and PD‐L1 as key nodes in the protective network formed by *A. muciniphila*. These statistical associations appear to be consistent with biological evidence. MMP‐10 has been identified as a key molecule driving atherosclerosis: gene knockout of MMP‐10 alleviates lesions in mice, and its high expression in human carotid plaques correlates with vulnerable plaque characteristics, such as increased calcification and thinning of the fibrous cap [49]. Additionally, genetic variants that enhance MMP‐10 directly increase the risk of large‐artery atherosclerotic stroke [50]. Meanwhile, PD‐L1 plays a central role in neuroprotection following ICH. It alleviates microglial M1 polarization and neuroinflammation by inhibiting the NF‐κB pathway [45]. Overexpression of PD‐L1 significantly reduces hematoma volume and improves prognosis, whereas blocking PD‐L1 reverses these protective effects [51]. In this context, *A. muciniphila*, commonly regarded as a member of the longevity‐associated bacteria, has been shown to benefit various chronic diseases, including neurodegenerative disorders, chronic kidney disease, and diabetes, by promoting balance within the gut microbiota [52]. It can be inferred that the protective effect of *A. muciniphila* B on CeVD development may arise from the release of MMP‐10 and PD‐L1.

This study has several strengths. First, our MR analysis was conducted at a more specific level of gut microbiota species compared with those in previous studies, facilitating the application of identified gut species for CeVD prevention and treatment. Second, we constructed a causal pathway from gut microbiota to CeVD by mediating inflammatory cytokines, which partially elucidates CeVD pathogenesis through mediation analysis. Third, the application of diverse MR methods along with sensitivity analyses enhanced the robustness of our findings.

However, we acknowledge some limitations in this study. First, because few IVs reached genome‐wide significance, the threshold for exposure IVs was set to 1e–05, but all IVs in this study demonstrated robust instrument strength through *F*‐statistic test. Second, the primary GWAS datasets comprised mostly European‐ancestry participants, necessitating cautious interpretation of trans‐ethnic applicability. Sensitivity analyses using cross‐population LD reference panels could partially address this limitation. Third, we did not validate against the larger MiBioGen consortium (*n* ≈ 18,000), as our prior study had already used that resource and we required the species‐level resolution provided by the FINRISK GWAS. The resulting species‐level gut microbiota signals may be more readily translatable and clinically actionable; we therefore plan to conduct a study in an independent population cohort to further verify their generalizability and clinical value.

## 5. Conclusions

In conclusion, through MR analysis, we explored the causal relationships between gut microbiota, inflammatory cytokines, and CeVD. Further mediation analysis revealed that inflammatory factors act as mediators in the pathway connecting gut microbiota to CeVD. These biomarkers have the potential to offer novel insights into the underlying mechanisms and facilitate targeted prevention and treatment strategies for CeVD.

## Author Contributions

Xinrui Wu contributed to the methodology, writing – review, funding acquisition, and conceptualization. Qi Li contributed to the methodology, formal analysis, and writing – original draft. Xinpeng Liu contributed to the formal analysis, writing – editing, and visualization. Xiang Deng contributed to the investigation, writing – editing, and funding acquisition. Houxiang Huang contributed to the investigation.

## Funding

This study was financially supported by the National Natural Science Foundation of China (Grant 82360665), the Natural Science Foundation of Hunan Province (Grants 2024JJ7406 and 2025JJ60925), the Scientific Research Project of Education Department of Hunan Province (Grant 24A0383), and the National Undergraduate Innovation Training Program Project (Grant S202510531068).

## Conflicts of Interest

The authors declare no conflicts of interest.

## Supporting Information

Additional supporting information can be found online in the Supporting Information section.

## Supporting information


**Supporting Information 1** Table S1. STROBE‐MR checklist. Table S2. MR analyses of gut microbiota on CeVD by different methods. Table S3. Tests for detecting horizontal and directional pleiotropy in forward MR analysis to detect the causal relationship between gut microbiota and CeVD. Table S4. Reverse MR analyses of CeVD on gut microbiota by different methods. Table S5. Tests for detecting horizontal and directional pleiotropy in reverse MR analysis to detect the causal relationship between gut microbiota and CeVD. Table S6. Multivariable MR analyses of gut microbiota on CeVD after adjusting confounding factors. Table S7. MR analyses of inflammatory cytokines on CeVD by different methods. Table S8. Tests for detecting horizontal and directional pleiotropy in forward MR analysis to detect the causal relationship between inflammatory cytokines and CeVD. Table S9. Reverse MR analyses of CeVD on inflammatory cytokines by different methods. Table S10. Tests for detecting horizontal and directional pleiotropy in reverse MR analysis to detect the causal relationship between inflammatory cytokines and CeVD. Table S11. Mediation effect of gut microbiota on CeVD via inflammatory cytokines.

**Supporting Information 2** Figure S1. Leave‐one‐out plots for the causal association between gut microbiota and IS in forward MR analyses. Figure S2. Leave‐one‐out plots for the causal association between gut microbiota and ICH in forward MR analyses. Figure S3. Leave‐one‐out plots for the causal association between gut microbiota and SAH in forward MR analyses. Figure S4. Leave‐one‐out plots for the causal association between inflammatory cytokines and IS in forward MR analyses. Figure S5. Leave‐one‐out plots for the causal association between inflammatory cytokines and ICH in forward MR analyses. Figure S6. Leave‐one‐out plots for the causal association between inflammatory cytokines and SAH in forward MR analyses.

## Data Availability

All analysis codes are publicly available at https://github.com/wuxr99/MR-and-Mediation-Analysis.
