# Supplementary material for: Investigating the Relationships Among Gut Microbiota, Inflammatory Cytokines, Cerebrovascular Diseases, and the Mediation Pathways
Source: Mediators Inflamm. 2026 Feb 23;2026:8623083. doi: 10.1155/mi/8623083 (PMC12929643; doi:10.1155/mi/8623083)
Supplement: Supplementary file 2 — Supporting Information 2 Figure S1. Leave‐one‐out plots for the causal association between gut microbiota and IS in forward MR analyses. Figure S2. Leave‐one‐out plots for the causal association between gut microbiota and ICH in forward MR analyses. Figure S3. Leave‐one‐out plots for the causal association between gut microbiota and SAH in forward MR analyses. Figure S4. Leave‐one‐out plots for the causal association between inflammatory cytokines and IS in forward MR analyses. Figure S5. Leave‐one‐out plots for the causal association between inflammatory cytokines and ICH in forward MR analyses. Figure S6. Leave‐one‐out plots for the causal association between inflammatory cytokines and SAH in forward MR analyses. [file MI-2026-8623083-s002.docx]

**Supplementary Figures 1-6**

**Investigating the Relationships among Gut Microbiota, Inflammatory Cytokines, Cerebrovascular Diseases, and the Mediation Pathways**

**Figure S1.** Leave-one-out plots for the causal association between gut microbiota and IS in forward MR analyses.

**Figure S2.** Leave-one-out plots for the causal association between gut microbiota and ICH in forward MR analyses.

**Figure S3.** Leave-one-out plots for the causal association between gut microbiota and SAH in forward MR analyses.

**Figure S4.** Leave-one-out plots for the causal association between circulating inflammatory cytokines and IS in forward MR analyses.

**Figure S5.** Leave-one-out plots for the causal association between circulating inflammatory cytokines and ICH in forward MR analyses.

**Figure S6.** Leave-one-out plots for the causal association between circulating inflammatory cytokines and SAH in forward MR analyses.


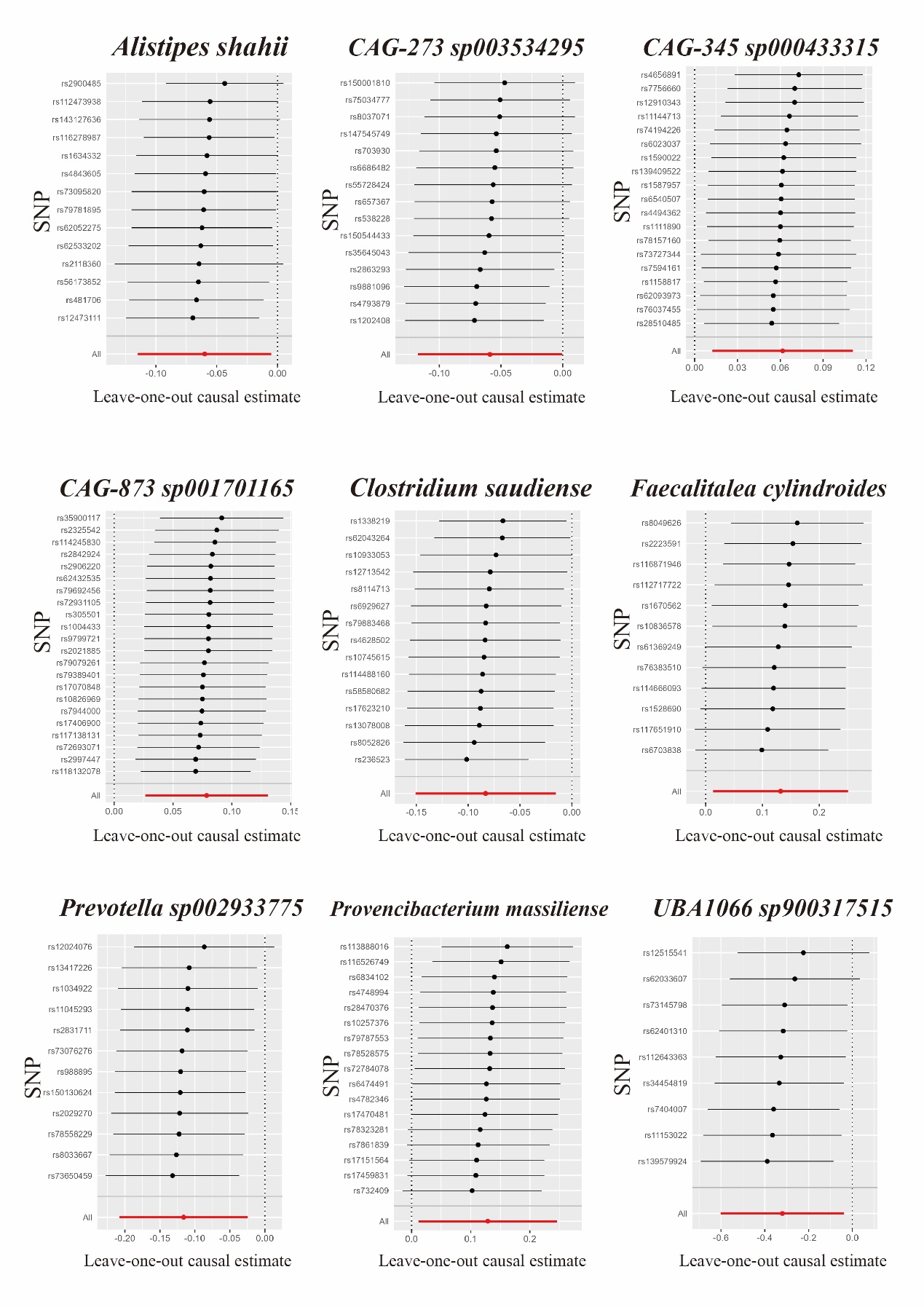


**Figure S1.** Leave-one-out plots for the causal association between gut microbiota and IS in forward MR analyses. The SNP in the figure represents the SNP sites involved in this MR analysis, and the horizontal black line represents 95% *CI* of the estimated causal effect value. Each black dot in the *CI* of each SNP is represented the effect value of MR causal estimation obtained when removing this SNP, using the remaining SNPs as IVs. IS, ischemic stroke; MR, mendelian randomization; SNP, single nucleotide polymorphism; *CI*, confidence interval; IVs, instrumental variables.


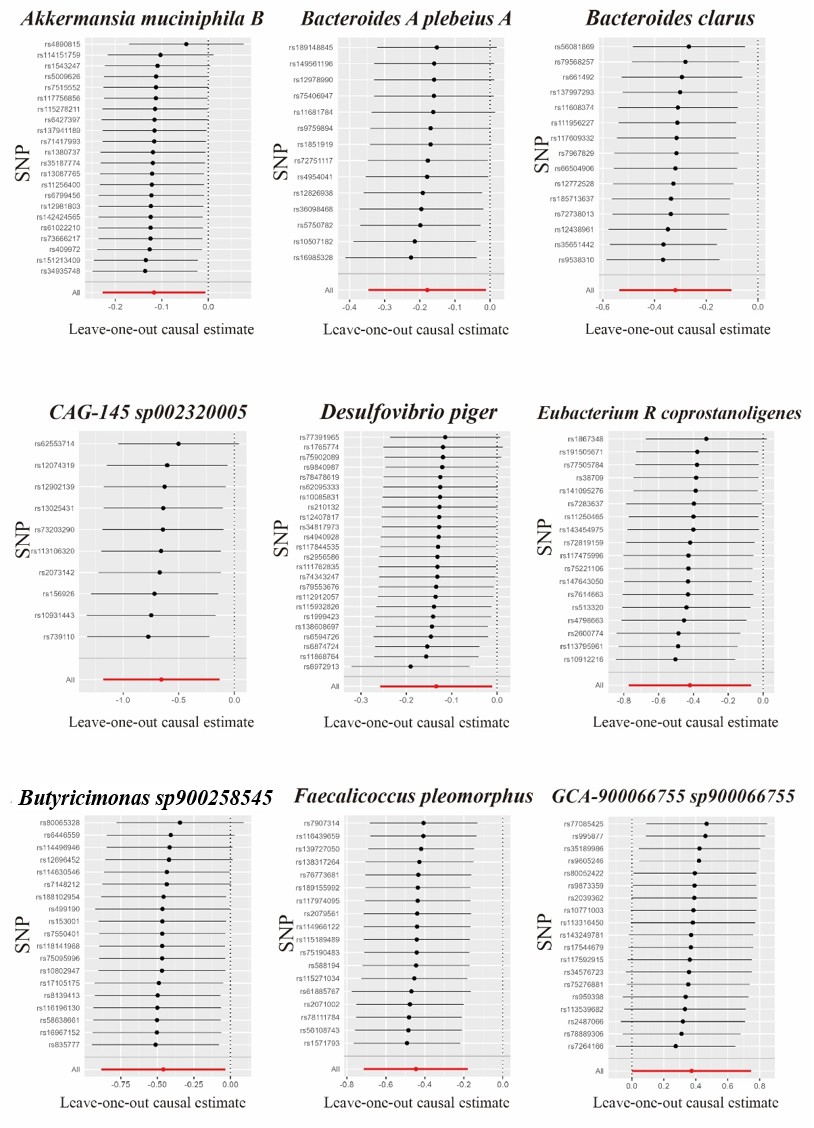


**Figure S2-1.** Leave-one-out plots for the causal association between gut microbiota and ICH in forward MR analyses. The SNP in the figure represents the SNP sites involved in this MR analysis, and the horizontal black line represents 95% *CI* of the estimated causal effect value. Each black dot in the *CI* of each SNP is represented the effect value of MR causal estimation obtained when removing this SNP, using the remaining SNPs as IVs. ICH, intracerebral hemorrhage; MR, mendelian randomization; SNP, single nucleotide polymorphism; *CI*, confidence interval; IVs, instrumental variables.


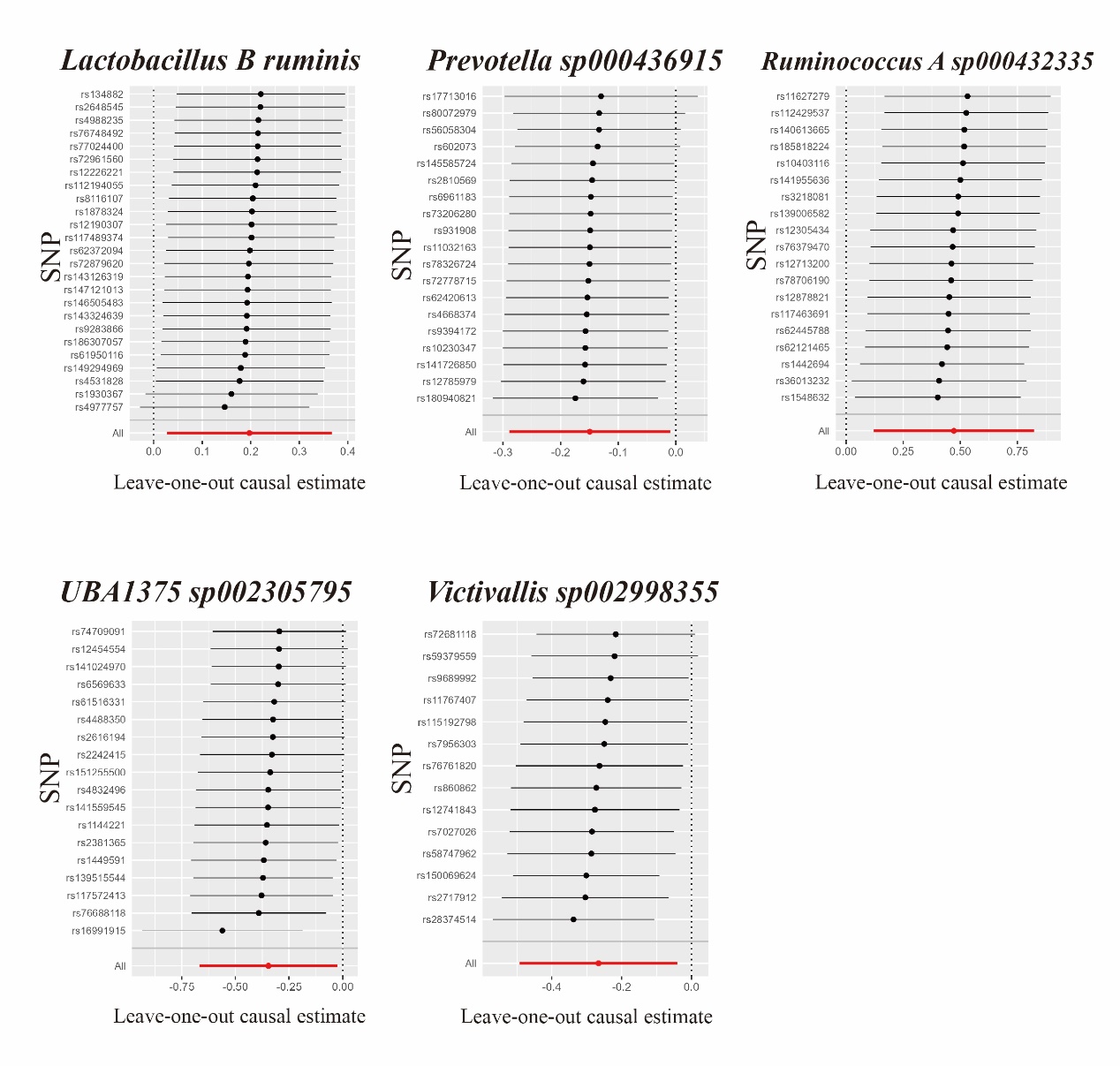


**Figure S2-2.** Leave-one-out plots for the causal association between gut microbiota and ICH in forward MR analyses. The SNP in the figure represents the SNP sites involved in this MR analysis, and the horizontal black line represents 95% *CI* of the estimated causal effect value. Each black dot in the *CI* of each SNP is represented the effect value of MR causal estimation obtained when removing this SNP, using the remaining SNPs as IVs. ICH, intracerebral hemorrhage; MR, mendelian randomization; SNP, single nucleotide polymorphism; *CI*, confidence interval; IVs, instrumental variables.


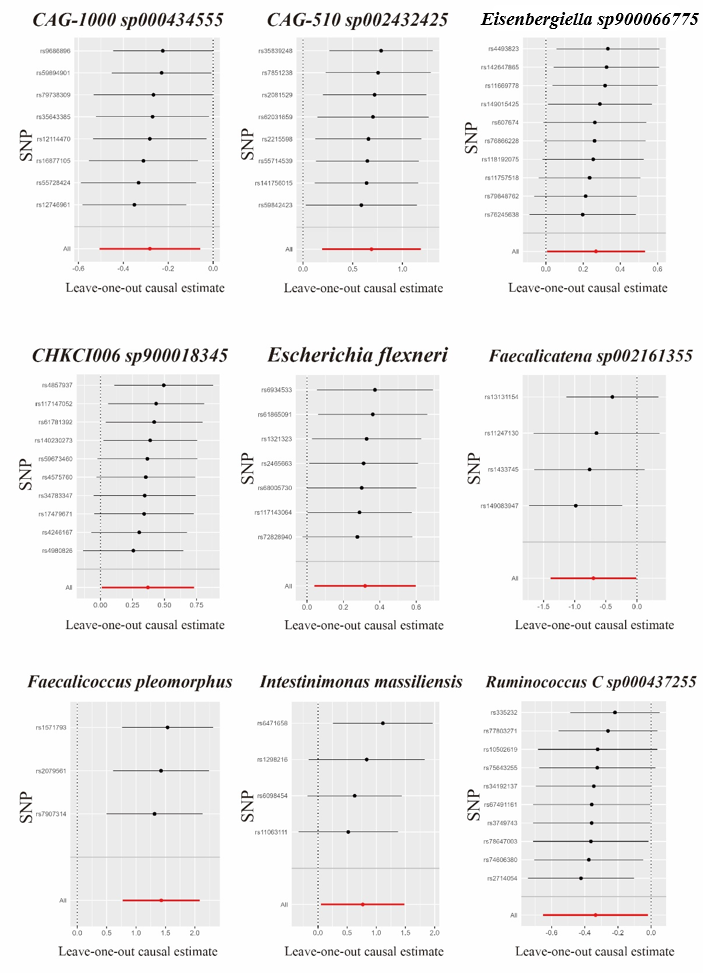


**Figure S3-1.** Leave-one-out plots for the causal association between gut microbiota and SAH in forward MR analyses. The SNP in the figure represents the SNP sites involved in this MR analysis, and the horizontal black line represents 95% *CI* of the estimated causal effect value. Each black dot in the *CI* of each SNP is represented the effect value of MR causal estimation obtained when removing this SNP, using the remaining SNPs as IVs. SAH, subarachnoid hemorrhage; MR, mendelian randomization; SNP, single nucleotide polymorphism; *CI*, confidence interval; IVs, instrumental variables.


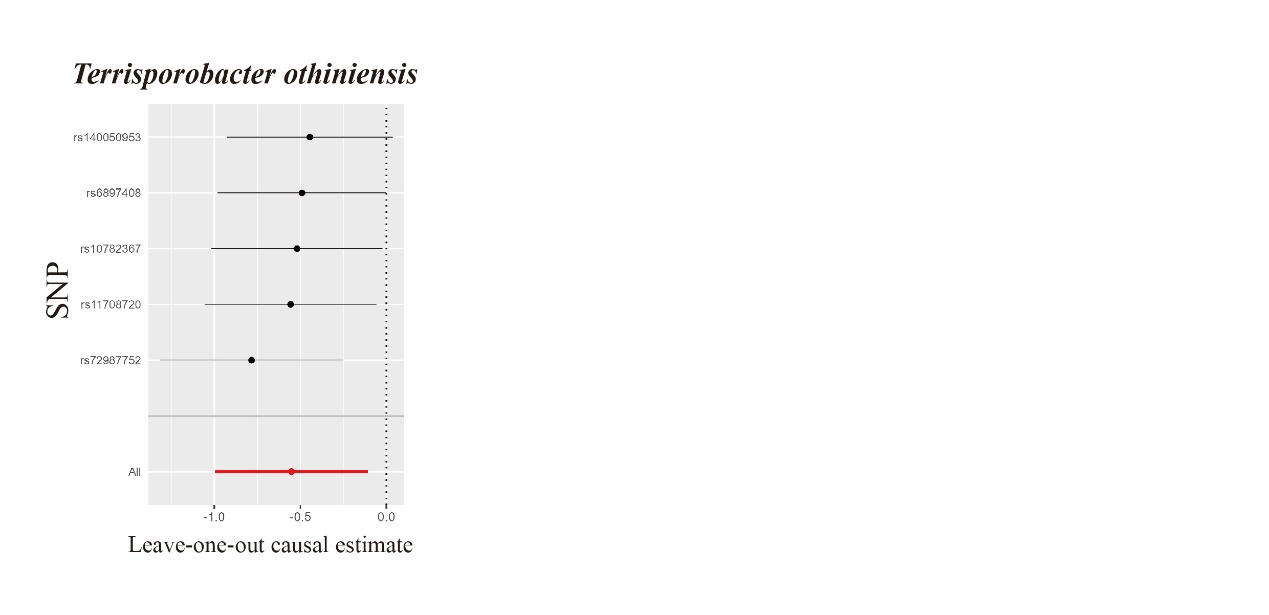


**Figure S3-2.** Leave-one-out plots for the causal association between gut microbiota and SAH in forward MR analyses. The SNP in the figure represents the SNP sites involved in this MR analysis, and the horizontal black line represents 95% *CI* of the estimated causal effect value. Each black dot in the *CI* of each SNP is represented the effect value of MR causal estimation obtained when removing this SNP, using the remaining SNPs as IVs. SAH, subarachnoid hemorrhage; MR, mendelian randomization; SNP, single nucleotide polymorphism; *CI*, confidence interval; IVs, instrumental variables.


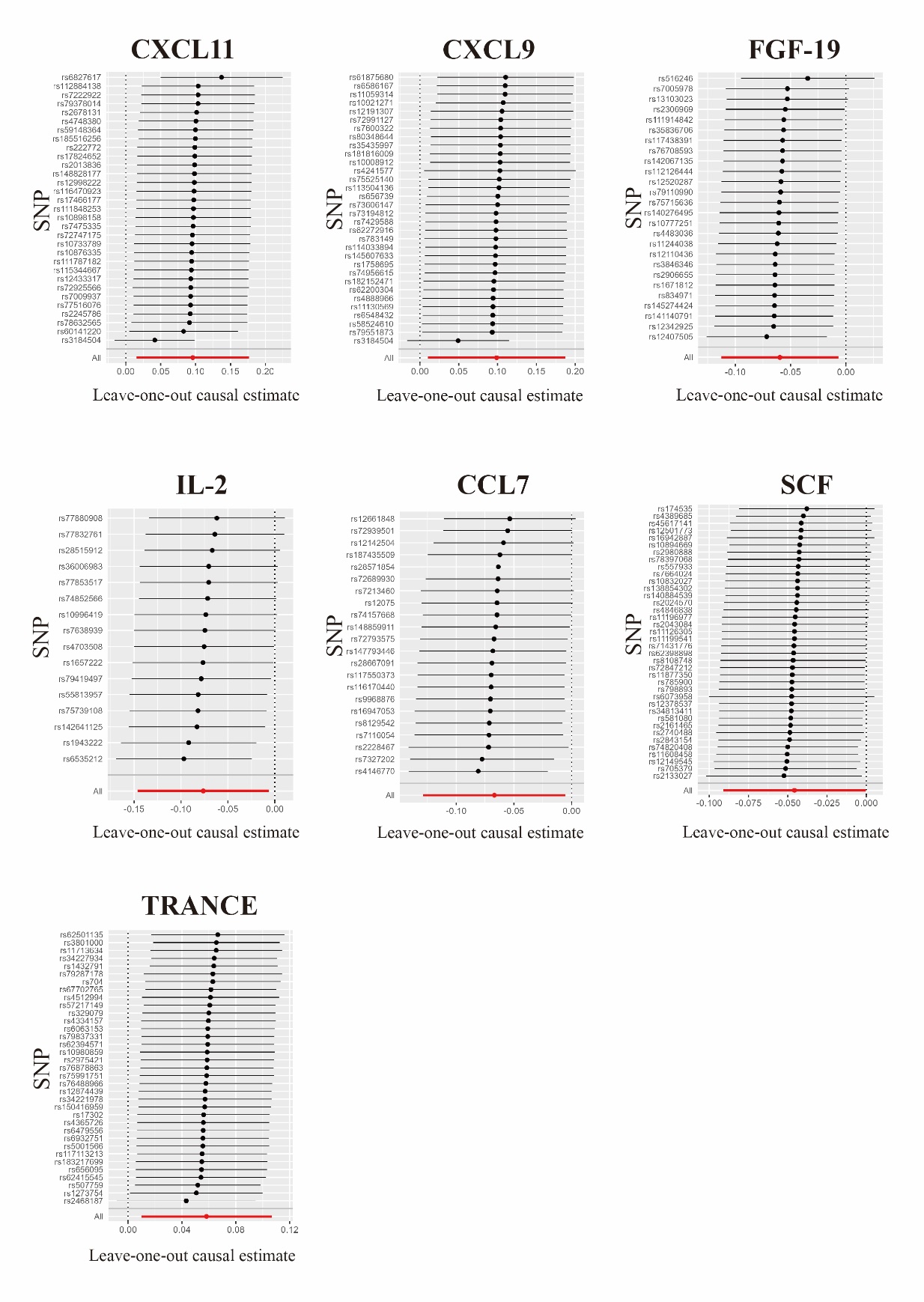


**Figure S4.** Leave-one-out plots for the causal association between circulating inflammatory cytokines and IS in forward MR analyses. The SNP in the figure represents the SNP sites involved in this MR analysis, and the horizontal black line represents 95% *CI* of the estimated causal effect value. Each black dot in the *CI* of each SNP is represented the effect value of MR causal estimation obtained when removing this SNP, using the remaining SNPs as IVs. IS, ischemic stroke; MR, mendelian randomization; SNP, single nucleotide polymorphism; *CI*, confidence interval; IVs, instrumental variables.


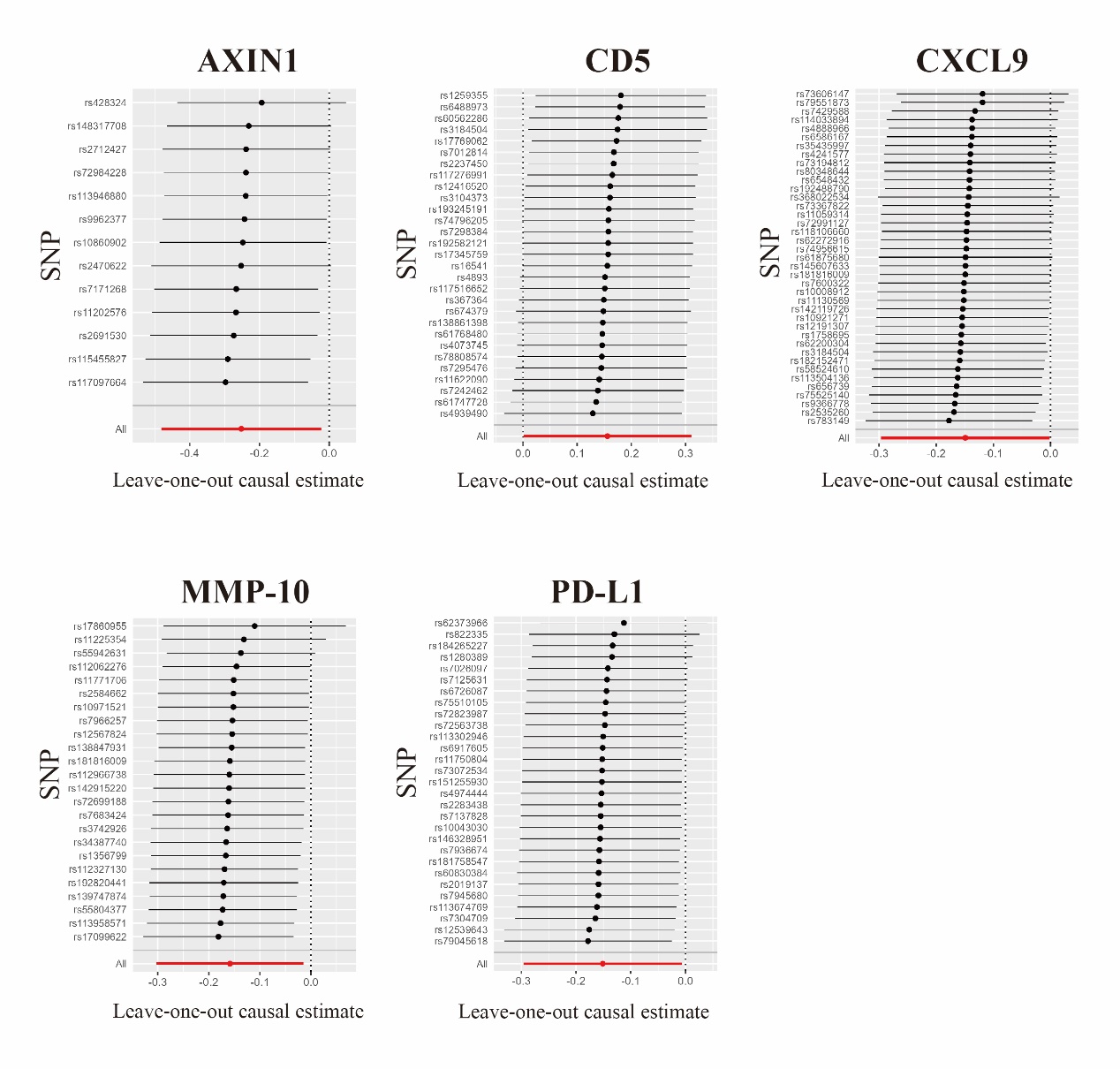


**Figure S5.** Leave-one-out plots for the causal association between circulating inflammatory cytokines and ICH in forward MR analyses. The SNP in the figure represents the SNP sites involved in this MR analysis, and the horizontal black line represents 95% *CI* of the estimated causal effect value. Each black dot in the *CI* of each SNP is represented the effect value of MR causal estimation obtained when removing this SNP, using the remaining SNPs as IVs. ICH, intracerebral hemorrhage; MR, mendelian randomization; SNP, single nucleotide polymorphism; *CI*, confidence interval; IVs, instrumental variables.


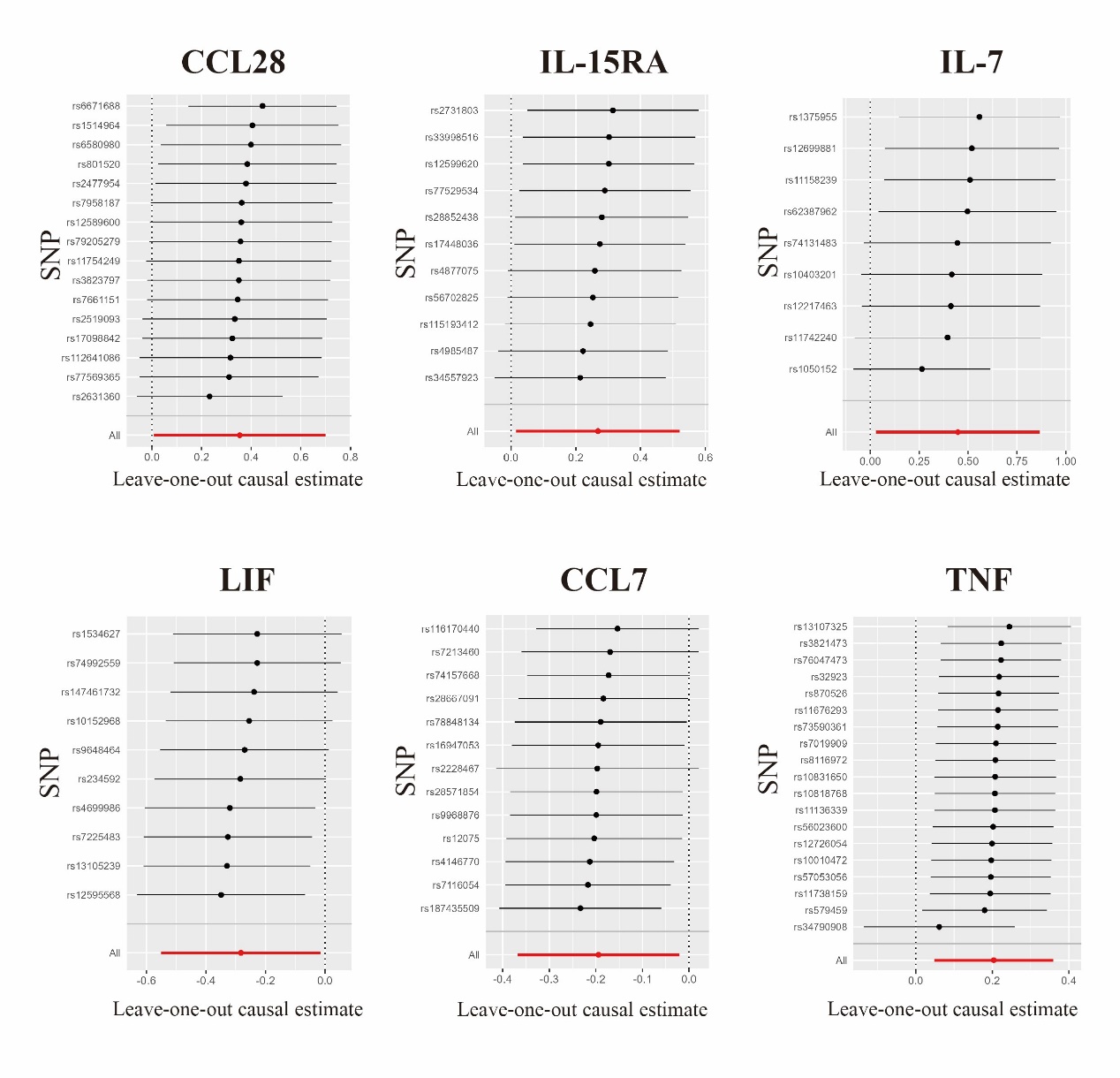


**Figure S6.** Leave-one-out plots for the causal association between circulating inflammatory cytokines and SAH in forward MR analyses. The SNP in the figure represents the SNP sites involved in this MR analysis, and the horizontal black line represents 95% *CI* of the estimated causal effect value. Each black dot in the *CI* of each SNP is represented the effect value of MR causal estimation obtained when removing this SNP, using the remaining SNPs as IVs. SAH, subarachnoid hemorrhage; MR, mendelian randomization; SNP, single nucleotide polymorphism; *CI*, confidence interval; IVs, instrumental variables.
